# European Summit on the Prevention and Self-Management of Chronic Respiratory Diseases: report of the European Union Parliament Summit (29 March 2017)

**DOI:** 10.1186/s13601-017-0186-3

**Published:** 2017-12-27

**Authors:** Peter W. Hellings, David Borrelli, Sirpa Pietikainen, Ioana Agache, Cezmi Akdis, Claus Bachert, Michael Bewick, Erna Botjes, Jannis Constantinidis, Wytske Fokkens, Tari Haahtela, Claire Hopkins, Maddalena Illario, Guy Joos, Valerie Lund, Antonella Muraro, Benoit Pugin, Sven Seys, David Somekh, Pär Stjärne, Arunas Valiulis, Erkka Valovirta, Jean Bousquet

**Affiliations:** 1 Department of Otorhinolaryngology, University Hospitals Leuven, KU Leuven, Louvain, Belgium; 2 Department of Otorhinolaryngology, Academic Medical Center, Amsterdam, The Netherlands; 3 Italian Member of the European Parliament, EFDD Group, Brussels, Belgium; 4 Finnish Member of the European Parliament, Brussels, Belgium; 5 Faculty of Medicine, Transylvania University, Brasov, Romania; 6 Swiss Institute of Allergy and Asthma Research (SIAF), University of Zurich, Davos, Switzerland; 7 Christine Kühne - Center for Allergy Research and Education (CK-CARE), Davos, Switzerland; 8 Upper Airways Research Laboratory, ENT Department, Ghent University Hospital, Ghent, Belgium; 9 Q4U Consultants Ltd, London, UK; 10 EFA - European Federation of Allergy and Airways Diseases Patients’ Associations, Brussels, Belgium; 11 1st Department of ORL, Head and Neck Surgery, Aristotle University, Thessaloníki, Greece; 12 Skin and Allergy Hospital, Helsinki University Hospital, Helsinki, Finland; 13 ENT Department, Guy’s and St Thomas’ Hospitals, London, UK; 14 Division for Health Innovation, Campania Region and Federico II University Hospital Naples (R&D and DISMET), Naples, Italy; 15 Department of Respiratory Medicine, Ghent University Hospital, Ghent, Belgium; 16 Royal National Throat, Nose and Ear Hospital, University College London Hospitals, London, UK; 17 Food Allergy Referral Centre Veneto Region, Department of Women and Child Health, Padua General University Hospital, Padua, Italy; 18 European Forum for Research and Education in Allergy and Airway Diseases (EUFOREA), Brussels, Belgium; 19 Lab of Clinical Immunology, Department of Immunology and Microbiology, KU Leuven, Brussels, Belgium; 20 European Health Futures Forum (EHFF), Isle of Wright, UK; 21 Rhinology Department of Otorhinolaryngology, Karolinska University Hospital, Stockholm, Sweden; 22 Vilnius University Clinic of Children’s Diseases and Public Health Institute, Vilnius, Lithuania; 23 European Academy of Paediatrics (EAP/UEMS-SP), Brussels, Belgium; 24 Department of Lung Diseases and Clinical Allergology, Univ. of Turku, and Allergy Clinic, Terveystalo, Turku, Finland; 25 MACVIA-France, Contre les MAladies Chroniques pour un VIeillissement Actif en France European Innovation Partnership on Active and Healthy Ageing Reference Site, Montpellier, France; 26 INSERM U 1168, VIMA: Ageing and Chronic Diseases Epidemiological and Public Health Approaches, UMR-S 1168, Université Versailles St-Quentin-en-Yvelines, Villejuif, Montigny le Bretonneux, France; 27 EUFOREA aisbl, 132, Ave. Brand Whitlock, 1200 Brussels, Belgium

**Keywords:** Advocacy, EUFOREA, Asthma, Mobile health technology, Allergy

## Abstract

On March 29, 2017, a European Summit on the Prevention and Self-Management of Chronic Respiratory Diseases (CRD) was organized by the European Forum for Research and Education in Allergy and Airway Diseases. The event took place in the European Parliament of Brussels and was hosted by MEP David Borrelli and MEP Sirpa Pietikainen. The aim of the Summit was to correspond to the needs of the European Commission and of patients suffering from CRD to join forces in Europe for the prevention and self-management. Delegates of the European Rhinologic Society, European Respiratory Society, European Academy of Allergy and Clinical Immunology, European Academy of Paediatrics, and European Patients Organization EFA all lectured on their vision and action plan to join forces in achieving adequate prevention and self-management of CRD in the context of Precision Medicine. Recent data highlight the preventive capacity of education on optimal care pathways for CRD. Self-management and patient empowerment can be achieved by novel educational on-line materials and by novel mobile health tools enabling patients and doctors to monitor and optimally treat CRDs based on the level of control. This report summarizes the contributions of the representatives of different European academic stakeholders in the field of CRD.

## Background

**Fig. 1:**
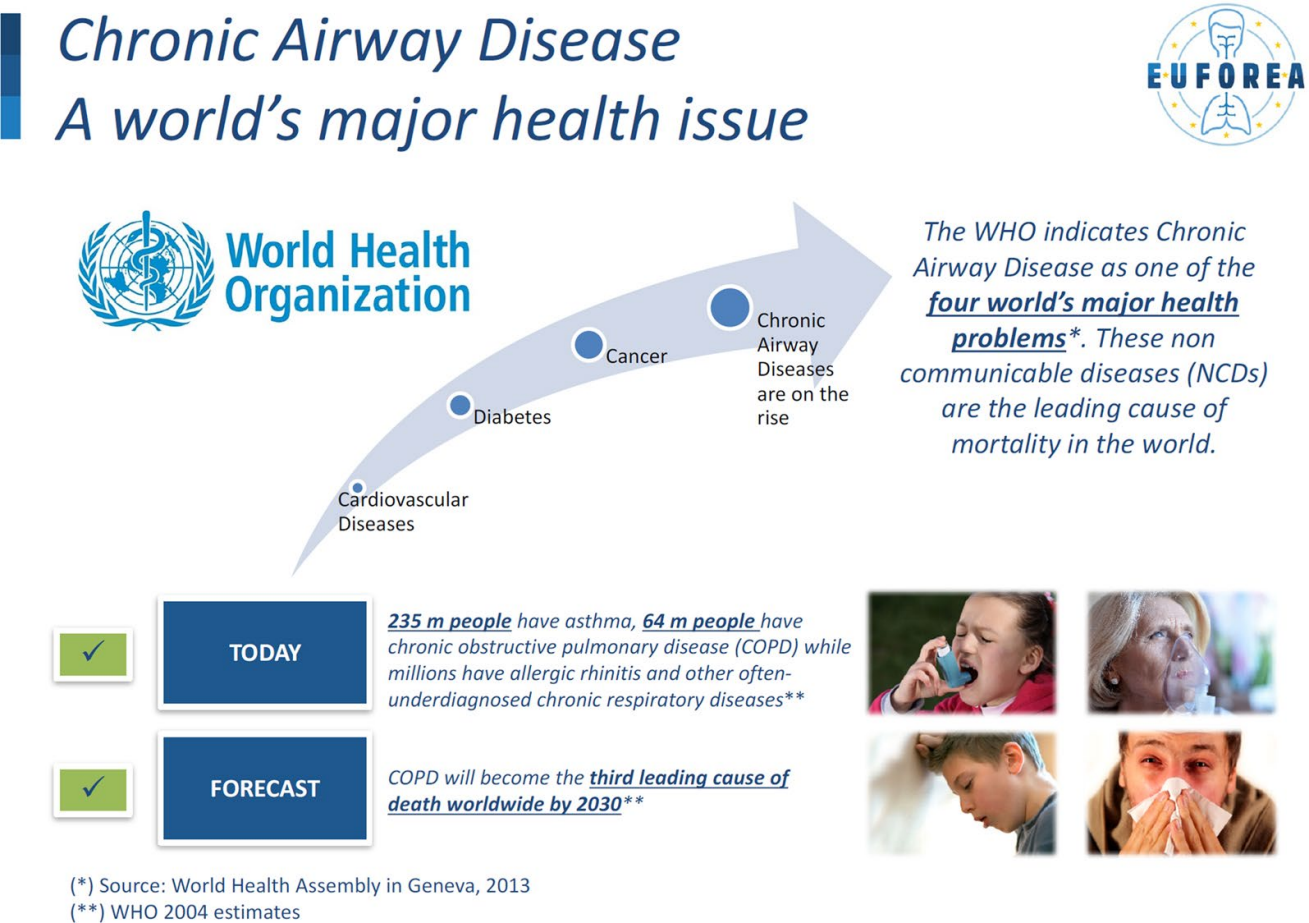
Chronic airway disease

The World Health Organization (WHO) rates Chronic Respiratory Diseases (CRD) as one of the 4 major chronic diseases of mankind (Fig. 1) [1]. CRD often start in early childhood and persist throughout the life cycle. They are a major cause of economic burden and largely impact on the general society. CRD represent a major global health problem leading to gender and social inequalities within and between countries [2]. CRD including allergies and chronic rhinosinusitis (CRS) are complex diseases intertwined with ageing. Asthma, CRS and allergic rhinitis (AR) are the most common non-communicable diseases in children and adults, and their prevalence and burden have increased in recent decades, reaching epidemic proportions [3–5]. The Polish Presidency of the Council of the European Union (EU) has therefore made prevention, early diagnosis and treatment of CRD a priority for the EU’s public health policy [6] to prepare the Conclusions of the Council, December 2, 2011 [7]. This was reinforced by the Cyprus Presidency of the EU Council [8]. In October 2015, a symposium on Precision Medicine in Allergy and Airway diseases was organized in the EU parliament with active participation of the Commissioner of Health Vytenis Andriukaitis [9]. An innovative integrated health system, built around systems and Precision Medicine and strategic partnerships, was proposed to combat CRD [10–13]:

- To better understand genetic determinants, environmental factors, molecular and cellular mechanisms underlying CRDs.
- To investigate social and economic factors leading to the onset, persistence and severity of CRDs.
- To phenotype and endotype patients, allowing implementation of the key pillars of Precision Medicine, i.e. personalized care, participation of the patient, prediction of success of treatment and prevention of disease.
- To develop unbiased and accurate biomarkers for multimorbidities, severity and follow-up of patients.
- To propose novel care pathways including multimorbidity and self-management strategies.
- To implement the principles of Precision Medicine into daily practice, in particular in primary care.
- To coach self-empowered patients through novel mobile health tools.
- To implement integrated care pathways with uniform information and treatment strategies provided by different care providers seen by patients.

A multidisciplinary practical platform with all stakeholders involved in CRD is currently missing. A symposium at the EU Parliament in October 2015 [9] proposed that the implementation of Precision Medicine into clinical practice may help to achieve the arrest of the epidemic of allergies and CRD. Participants underscored the need for optimal patient care in Europe, supporting joint action plans for disease prevention, patient empowerment, and cost-effective treatment strategies [9]. In the meantime, EUFOREA elaborated a European consensus report on the guidance of clinicians into the implementation of the principles of PM in daily practise [13].

**Fig. 2:**
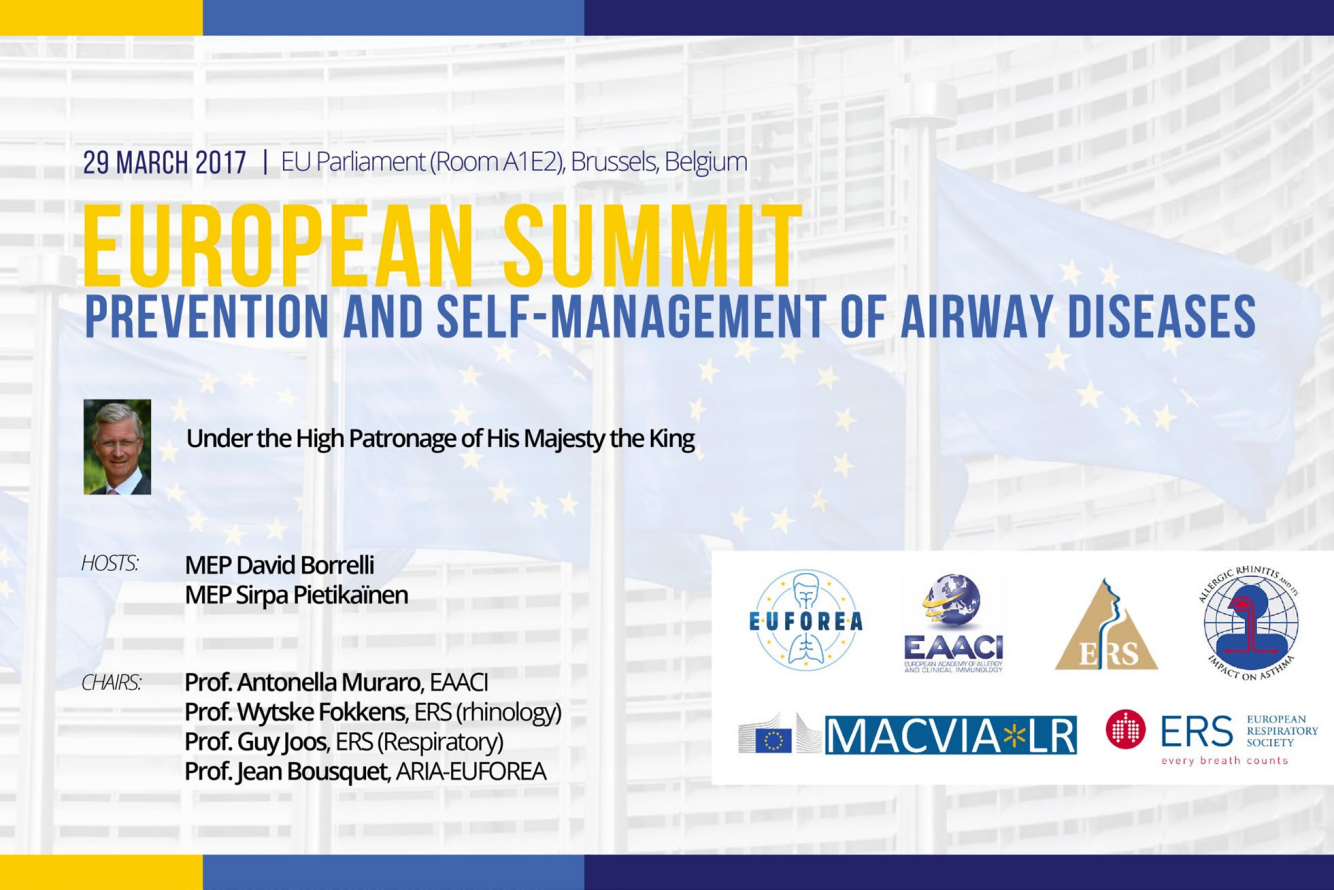
Programme announcement of EU Summit

**Fig. 3:**
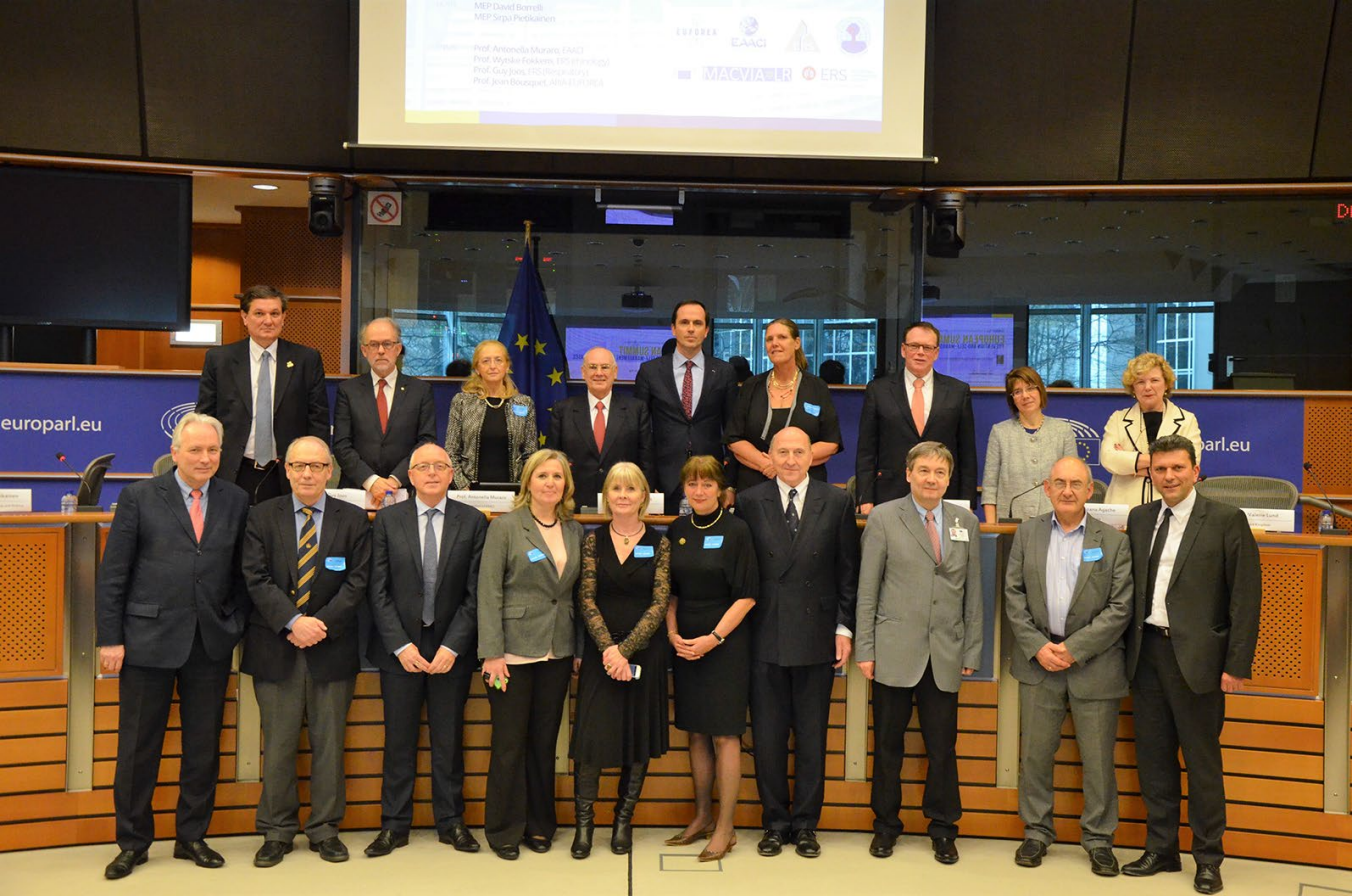
Faculty portrait

The European Union Parliament Summit on March 29, 2017 aimed at providing practical approaches on the prevention and self-management of CRD, proposing care pathways implementing emerging technologies for predictive medicine centred around the patient (Figs. 2, 3). The Summit involved the active contribution of the scientific societies and patients’ organisations, allowing a discussion on the innovative EUFOREA approaches to move forward on the prevention and self-management of CRD.

## The vision, mission and objectives of EUFOREA

EUFOREA is an international non-profit organisation forming an alliance of all stakeholders from national and international organizations, institutions, and agencies working towards the common vision of preventing and improving the burden of asthma, allergic airway diseases and CRS. EUFOREA proposes to reduce the preventable and avoidable burden of morbidity and disability due to CRD by means of a multidisciplinary practical approach with all stakeholders at national, regional and global levels. Its aim is for populations to reach the highest attainable standards of health and productivity at every age and for CRD to no longer present as a barrier to well-being or socio-economic development. Given the novel scientific data on the prediction and prevention of allergic and airway diseases, the cost of inaction is unacceptable and there is an urgent need to undertake integrated actions in Europe, joining forces in a multi-specialty way with all stakeholders.

The main objective of EUFOREA is to initiate a comprehensive approach to prevent and fight CRD in upper and lower airways via the following strategies:

- To better educate the patients, the (para)medical community and the public
- To develop innovative care pathways including the key principles of Precision Medicine, i.e. prevention, personalized care, prediction and participation
- To empower the patients at the centre of the strategy, and to promote self-management and primary care
- To educate patients and health care providers on the optimal care pathways for CRD, taking into account 4 approaches: prevention, prediction, participation and personalized care
- To make recommendations of simple strategies for chronic airway disease management using emerging technologies for predictive medicine
- To promote active and healthy ageing
- To improve the work productivity of chronic airway disease sufferers
- To improve the well-being of chronic airway disease sufferers
- To reduce health and social inequities
- To support academic research in Europe in the field of CRD through a unique research support platform.

The added value of EUFOREA is to develop a unique strategic partnership for the prevention and control of CRD in order to tackle all components of prevention and to combat CRD from basic science to policies. EUFOREA provides a network through which collaborating parties can combine their strengths, thereby achieving major results that no one single partner could obtain alone. EUFOREA is meeting the need for a *pan-European multi-stakeholder platform* for the development of optimal and integrated care pathways for CRD, through a coordinated research and education activity portfolio. The EUFOREA platform unites educational and research initiatives of all relevant stakeholders dealing with CRD through representatives of their official organizations: Primary Care physicians, Paediatricians and Allergologists via EAACI (European Academy of Allergy and Clinical Immunology), Respiratory physicians via ERS (European Respiratory Society), ENT doctors via ERS (European Rhinology Society), Allergy specialists of the ARIA (Allergic Rhinitis and its Impact on Asthma) expert group, Rhinologists of the EPOS expert committee, Patient organizations via EFA (European Federation of Allergy and Asthma Patient Organization), and Pharmacists via PGEU (Pharmaceutical Group at the European Parliament). The EUFOREA platform is unique given the multi-specialty nature and high academic profile of the multi-specialty advisory board taking part in the educational and clinical research activities of EUFOREA.

EUFOREA attempts to improve coordination between existing EU, governmental and non-governmental programmes to avoid duplication of efforts and wasting of resources.

## Action plan of EUFOREA

1. *ARIA* Care pathways implementing emerging technologies for predictive medicine in rhinitis and asthma across the life cycle

The Allergic Rhinitis and its Impact on Asthma (ARIA) initiative commenced during a WHO workshop in 1999 and was developed and implemented by the WHO Collaborating Centre on Asthma and Rhinitis [14, 15]. The initial goals were:

- To propose a new AR classification,
- To promote the concept of multi-morbidity in asthma and rhinitis, and
- To develop guidelines with all stakeholders that could be used globally for all countries and populations, in particular developing countries.

ARIA—disseminated and implemented in over 70 countries globally [16]—is now focusing on the implementation of emerging technologies for individualized and predictive medicine. MASK (MACVIA (*Contre les MAladies Chroniques pour un VIeillissement Actif*) [17]—ARIA Sentinel NetworK) [18] uses mobile technology to develop care pathways for the management of rhinitis and asthma by a multi-disciplinary group and by patients themselves. An App (Android and iOS) is available in 21 countries and 16 languages. It uses a visual analogue scale to assess symptom control and work productivity as well as a clinical decision support system. It is associated with an inter-operable tablet for physicians and other health care professionals. The scaling up strategy uses the recommendations of the European Innovation Partnership on Active and Healthy Ageing. The aim of the novel ARIA approach is to provide well-being and an active and healthy life to rhinitis sufferers, whatever their age, sex or socio-economic status, in order to reduce health and social inequalities incurred by the disease [19].

2. *EUFOREA mobile application ‘mySinusitisCoach’* CRS as a model for optimal patient coaching and empowerment

CRS is a common disease but a stratification strategy is needed to provide a satisfactory management and to avoid unnecessary surgical interventions. MySinusitisCoach is being developed by EUFOREA, following a unique strategy with the following phases of development:

1. European CRS patient days to explore the needs and obstacles in current CRS care, including feedback on the mySinusitisCoach prototype using emerging technologies and the proposal of CRS interactive educational materials.
2. European meeting to align the patients’ needs and KOL vision on the mySinusitisCoach prototype (Fig. 4), with feedback on the unique educational and coaching strategy of the CRS patients by mySinusitisCoach.

**Fig. 4:**
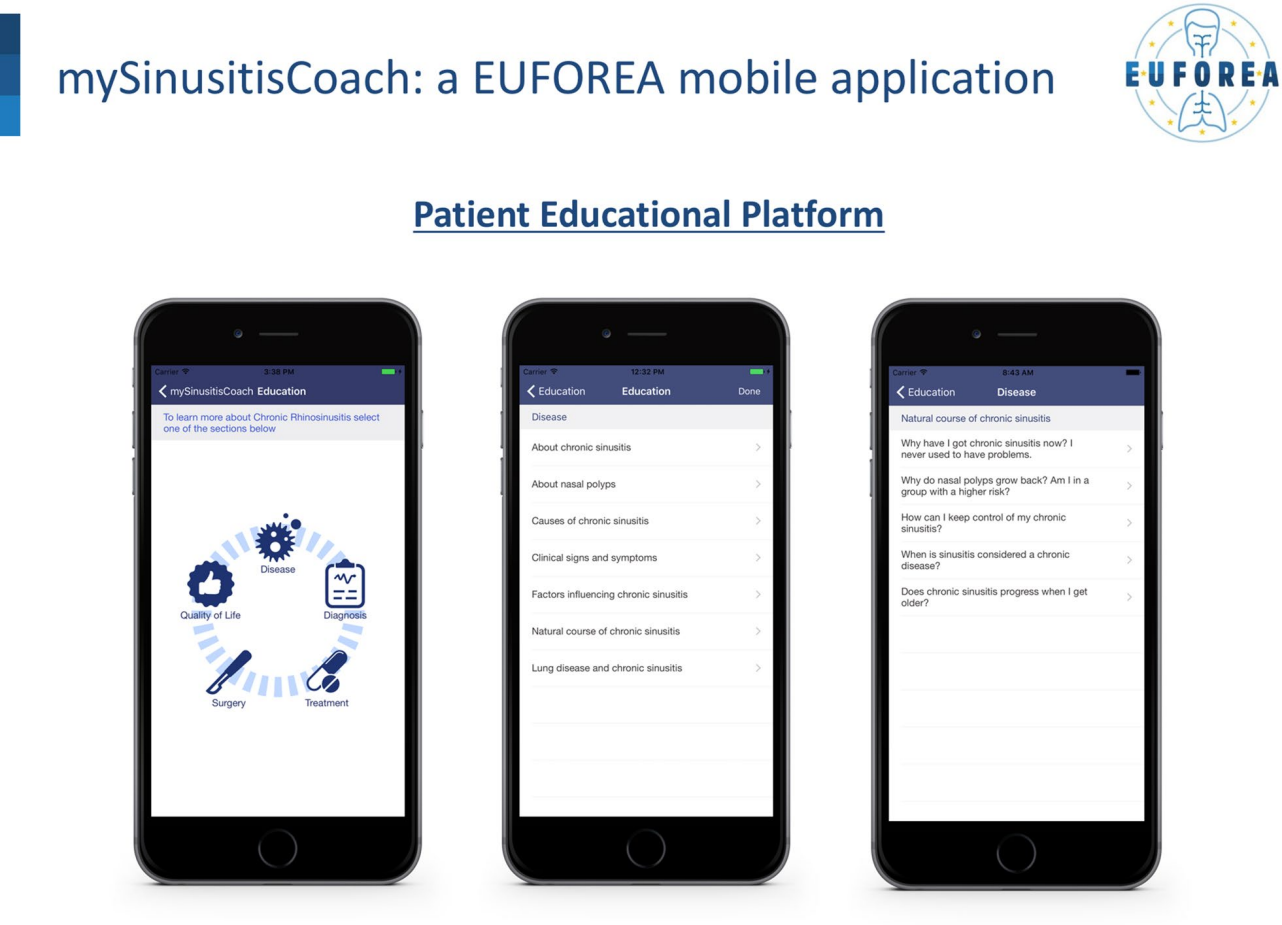
EUFOREA ‘mySinusitisCoach’
3. Preparation of the implementation of mySinusitisCoach in several regions in Europe with the support of local governmental authorities.
4. Analyses of the data generated by mySinusitisCoach, showing socio-economic value, secondary and tertiary prevention of CRD
5. Deployment of mySinusitisCoach in Europe, with data on real-life burden and cost of disease, (in)effective current treatment strategies, and demonstration of the major benefit of the instalment of mySinusitisCoach for the patient and the society.
6. EUFOREA Educational Platform: a practical approach

**Fig. 5:**
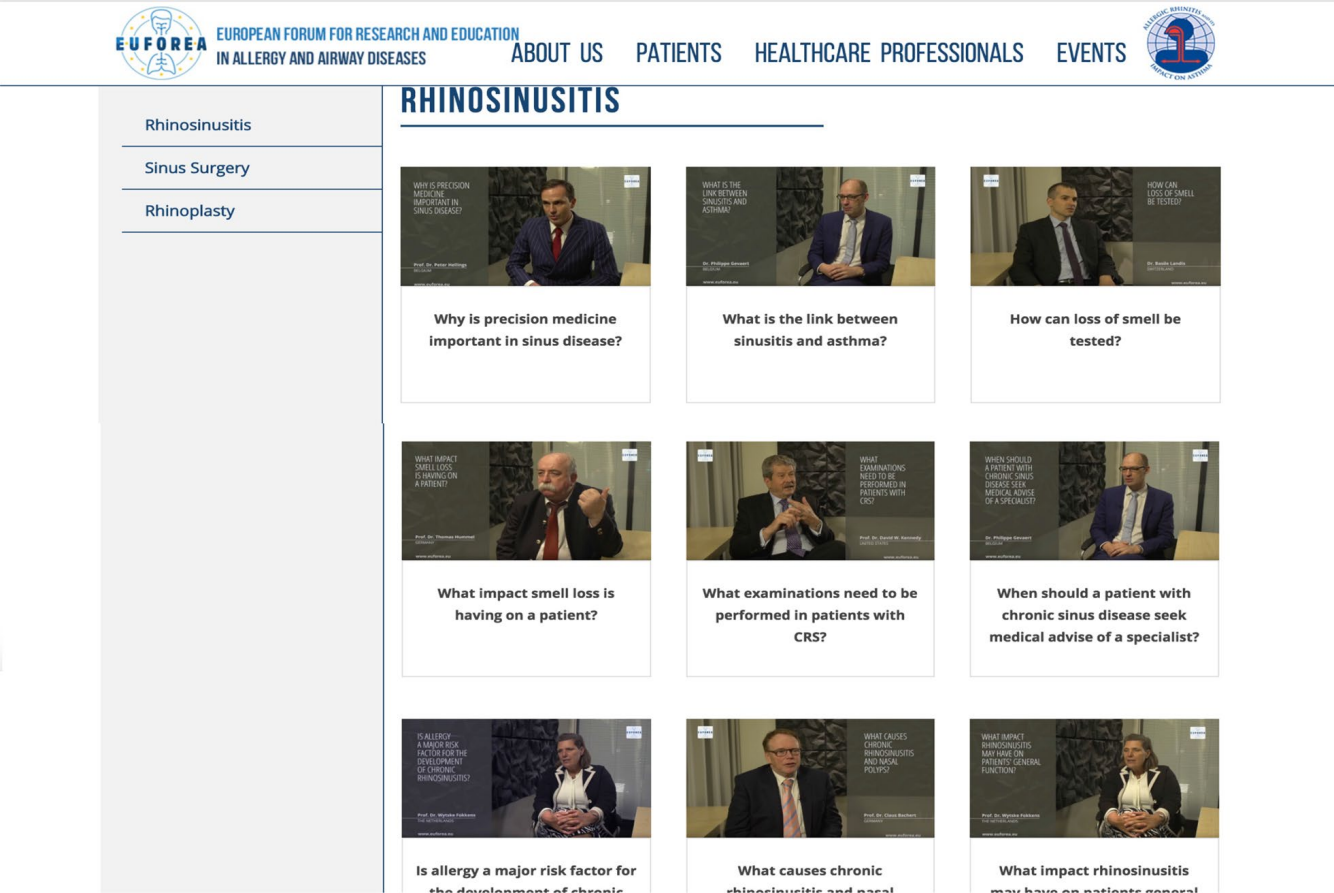
EUFOREA online Patient Educational Platform

Patient education is the process by which health professionals and others impart information to patients and their caregivers that will alter their health behaviour or improve their health status. Patient education for patients with chronic upper airway disease is one of the mainstays of EUFOREA (Fig. 5). European experts made a list of questions asked in their daily practice. Subsequently, interviews with small group of patients, individually and in groups, about their information needs were organized for understanding the patients’ needs. A large web-based interview about information needs was then elaborated. Patients indicated that they needed more information at all stages of their disease and that they were not satisfied with the information available on the internet or from other sources. Patients were mostly interested to receive their information by means of a website or an App. Furthermore, they were very enthusiastic about the possibility of consulting a sinusitis coach (trained healthcare professional) by mail, chat or telephone. The EUFOREA website already contains the first series of patient information.

4. EUFOREA Respiratory Research Platform for defining the RESEARCH NEEDS AND PRIORITIES in Europe

In November 2016, brainstorming sessions were held during the European Rhinology Research Forum (ERRF) on the research needs and priorities in the field of CRD with active participants from all over Europe. The major unmet needs and research priorities in the field of allergic rhinitis and Chronic Respiratory Diseases were highlighted from the perspectives of the patients and clinicians. This meeting resulted in the definition of the research priorities in the field of CRD and the joint action plan to meet these priorities [20]. A follow-up meeting is planned in the Royal Academy of Medicine of Belgium in Brussels on November 9 and 10, 2017, involving basic researchers, clinicians, representatives of patient organizations, and representatives of the industry and health authorities.

## The role of scientific societies

European scientific societies are crucial in the management of CRD. They provide evidence-based, state-of-the art guidance for health practitioners by means of publishing research and knowledge including clinical practice guidelines, statements and technical standards as well as by providing continuing medical education (CME). In addition, the large-scale annual or bi-annual congresses aim at transferring the latest scientific data to the clinicians. They bridge the gap between science and policy and advocate for policies committed to protecting public health. Scientific societies need to provide evidence-based input to ensure that airway diseases and their management is at the centre of policy decisions including air quality, European medical research, tobacco control and other preventative approaches such as occupational health issues. In conjunction with EFA, EAACI is currently launching a political Call to Action on Allergy and Asthma, with the support of the Interest Group on Allergy and Asthma of the European Parliament, led by the Finnish MEP Sirpa Pietikainen.

## Prevention of allergy and asthma in Finland

In the early 1990s, the new paradigm of asthma as an inflammatory disease was implemented into practice by the *Finnish Asthma Programme 1994*–*2004*. This improved care and cut costs both for individual patients and society [21]. New information of immune development in modern, urban societies has challenged the conventional thinking of allergy prevention. *The Finnish Allergy Programme 2008*–*2018* was initiated to turn avoidance strategy into tolerance strategy, and to take a step from treatment to prevention [22, 23]. Focusing on severe allergies and emphasizing allergy health rather than mild problems also targeted the use of health-care resources more efficiently. Revisiting of the asthma and allergy paradigms has led to actions relevant to society and health-care as a whole. In the Finnish society, burden of these conditions has started to decline [24]. The experience encourages medical communities and societies to lessen disability and costs caused by allergy and asthma and to improve public health.

## Therapeutic strategies to prevent asthma

Asthma often starts early in life and tends to persist across the life cycle [2, 25]. Thus, asthma prevention is a continuum from conception (or even before) to old age, integrating the complex network of risk factors (genome, exposome and disease endotypes and phenotypes) into a detailed roadmap for action [26]. Today the projection of children’s diseases in adulthood is the key target for research and prerequisite of effective prevention as well as healthy ageing [27]. Wheezing is the major problem in both developed and developing countries affecting up to 50% of children under the age of 6 years. Even transient early wheeze is not only characterized by lower lung function but is also associated with chronic obstructive pulmonary disease in late adulthood [28]. An important work point is to protect children from active and passive smoking. Speed networking and new technologies based e-research allow different projects to be integrated as well as the building of roads to modern paediatric respiratory medicine in EU countries [29].

A step-wise pathway is proposed: the tomorrow action plan relies on the multi-strategy approach focused on primary prevention and including the targeted hygiene concept. The 5-year programme relies on the wide use of health information technology translating vast amounts of “real-world” data unbiased by any pre-selection criteria into real-time clinical decision support at the point of care and harmonised disease management based on quality criteria [30]. The 10-year strategy integrates the multi-strategy approach with big data into personalised prevention plans [31]. Global, multi-discipline partnerships, rethinking healthcare, agreeing and implementing a fair balance between research and political priorities are all key points for an efficient asthma prevention strategy [32].

### Immunologic rationale for prevention of CRD

Dysregulated immune system, exposure to pollutants, irritants and allergens at home or to professional irritants in the work environment [33, 34], schools and workplace, genetic factors, and respiratory infections all play a role in the increased prevalence of CRD [35, 36]. Tobacco smoke is a key factor in the development and progression of CRD. Activation of the immune system cells and their interaction with tissue cells followed by chronic inflammation is a key mechanism in their pathogenesis. A defect in allergen tolerance is the cause of allergic diseases and is based on immunologic mechanisms. Recently, leakiness in the epithelial barrier is a major finding in all Chronic Respiratory Diseases such as asthma, AR and CRS [37, 38]. Several strategies are being developed to prevent respiratory diseases, including the restoration of barrier deficiency. Identification of risk groups and early intervention to restore the barrier function are essential for successful prevention strategies [39–41].

### A specific novel promising asthma prevention strategy resulted from the national survey in the United Kingdom

A prospective audit including more than 3 000 UK CRS surgical patients and assessing outcomes over a 5 year period showed significant improvement in the vast majority of patients [42]. Patients were then stratified by onset of symptoms to surgery into early (< 1 years), mid (1–5 years) and late (> 5 years) cohorts. This showed that the percentage change from baseline of CRS outcomes (SNOT22 scores) was greater in the early than in the late cohort at all time points (*p* < 0.005 at 1 years) when other demographic factors and extent of surgery were controlled [43]. Asthma prevalence was significantly higher in the late group, suggesting that earlier sinus surgery is associated with better outcomes in both the upper and lower respiratory tract, altering natural history and preventing co-morbidities.

### Strategies for broad implementation of effective self-care

PROSTEP (Promotion of self-care in chronic diseases in the EU) is a tender designed to explore the added value of self-management in chronic diseases. It benefits from a thorough analysis of patient empowerment, including the relationship between self-management, joint decision-making and health literacy. PROSTEP stemmed from the EMPATHiE project (Empowering patients in the management of chronic diseases http://www.eu-patient.eu/whatwedo/Projects/EMPATHiE/), the first of two previous tenders, and from the experience of a platform of experts addressing the issues around the promotion of self-care in minor and self-limiting conditions (the PiSCE project). The overall requirements of this tender were (1) to conduct a study (consisting of a literature review and cost–benefit analysis) and (2) to set up a platform of experts in self-care in the field of chronic diseases to explore and propose possible methods of promotion of self-care for chronic diseases, taking into account previous and on-going policy work in this field such as the work of the CHRODIS JA. The 2-year project kicked off in January 2016.

### Patient’s empowerment

The European Federation for Allergy and Airways Diseases Patients’ Associations (EFA) has launched different projects at EU level to empower patients. They are built on three pillars: education, self-management and advocacy. One of the projects, Hey Ya, aims at improving health literacy in adolescent patients via raising self-awareness, confidence and attitude. This is particularly important in teenagers in whom poor adherence due to reduced health literacy is one of the major contributors to uncontrolled allergic airway disease. Secondly, the My Air Coach project was launched by the help of the Horizon 2020 Research and Innovation framework programme with the aim to develop a patient-friendly, sensor-based tool to collect clinical, environmental and behavioural data relating to the patient. Via self-management, it is a powerful tool for the prevention of complications. mHealth technology, jointly with patients’ personal experience, are important for raising self-efficiency. Both Hey Ya and My Air Coach are important tools for improving teenagers’ approach toward the diseases. Lastly, EFA supports and actively participates in advocacy campaigns at EU level. Recently, the Written Declaration on Chronic Respiratory Diseases was closed and signed by 115 MEPs. A new ‘United Action for Allergy and Asthma’ was launched in the EU parliament in April 2017 to improve public policies. It addresses allergy and asthma and supports patients’ rights.

### Technology transfer in rhinitis and asthma

The European Innovation Partnership on Active and Healthy Ageing (EIP on AHA) includes 74 Reference Sites. Scaling up is an essential component of the Action Plan B3 of the EIP on AHA (chronic diseases and remote monitoring) [27, 44]. A transfer innovation from an App developed by the MACVIA-France EIP on AHA reference site (*Allergy Diary)* to other reference sites will compare the phenotypic characteristics of rhinitis and asthma in adults and old age people using validated ICT tools (*Allergy Diary* [19] and CARAT: Control of Allergic Rhinitis and Asthma Test [45]) in 25 Reference Sites or regions across Europe. The aim is to better understand, assess the burden, diagnose and manage rhinitis and asthma in old age people.

## Conclusions

The high prevalence and major socio-economic impact of CRD require an inter-academic and multi-stakeholder approach for the successful implementation of prevention strategies leading to cost savings and reduction of burden. EUFOREA will continue its’ mission to call for action by all stakeholders to implement Precision Medicine as the tool to arrest the epidemic of CRD. In Europe, there is an urgent need to join forces in the education of patients and medical care providers on prevention strategies, and to call for political action supported by all European academic stakeholders involved in the care of CRD.
